# Analysis of Functional Arch Support Insoles on the Biomechanics and Performance in Right-Forward Lunging Step of Badminton Players

**DOI:** 10.3390/ijerph191811210

**Published:** 2022-09-07

**Authors:** Hung-Wen Chen, Hsien-Te Peng, Yan Wei

**Affiliations:** 1 Graduate Institute of Coaching Science, Chinese Culture University, Taipei 11114, Taiwan; 2 Department of Physical Education, Chinese Culture University, Taipei 11114, Taiwan

**Keywords:** sports performance, lower extremity stability, anterior cruciate ligament injury, ground reaction force, kinematics, kinetics

## Abstract

The purpose of this study was to examine the differences in biomechanical parameters and sports-specific performance of lower limbs between arch support insoles (ASI) and flat insoles (FLI) when performing net strides. After installing the MVN IMU system, 18 college badminton team members were asked to take the following tests: (1) Consecutive net stride tests; (2) Six-point footwork tests; (3) Retrieve/stroke the ball at the left and right net; (4) Smash and retrieve/stroke the ball at the net; (5) Smash at the front and back crossover step. The joint angle of the lower limbs and ground reaction force during the support phase was collected. The results demonstrated that the peak right hip flexion angle was significantly greater with ASI than FLI (63.09 ± 10.70; 60.08 ± 13.82; *p* = 0.028), while the peak right foot inversion angle was significantly smaller with ASI than FLI (20.68 ± 7.87; 23.85 ± 8.11; *p* = 0.013). The principal conclusion was that the arch support insole avoids the decrease in the hip flexion angle and the increase in the foot inversion angle during the net stride tests.

## 1. Introduction

During athletic competition or training, athletes have a 60% chance of sustaining lower extremity injuries, such as ankle ligament sprains, knee injuries, and thigh tendon strains, with knee injuries being the most common, usually a cruciate ligament injury [1]. In addition, meniscus injuries, iliotibial band syndrome, and popliteal tendinitis are also common knee injuries [2]. Reducing the occurrence of sports injuries and minimizing the impact on athletic performance is a problem faced by all sports teams.

Forward stride is the most frequently used movement in badminton [3], allowing the athlete to quickly stop the body’s movement and stabilize the body in preparation for the next movement [4]. This movement allows the athlete to accelerate and decelerate and change direction in a short period [5,6,7]. It has been shown that speed or directional changes are considered responses to stimuli that involve body activation, directional changes, or acceleration and deceleration, which are closely related to body control [8]. Athletes on the court, who need to be aware of the situation and the position of the opponents at all times, are often faced with sudden changes of direction, such as cutting and forward stride, which may lead to less knee flexion and greater knee abduction. When athletes are unable to fully concentrate on their movements, they may be at greater risk of ACL injury [9]. Most lower extremity injuries in badminton players occur mainly to the ankle and knee, and ankle sprains usually occur during jump landings in backward and sideways movements or forward strides [10,11]. As such, forward stride in badminton may affect abrupt stop and directional change due to the instability of the lower limbs.

The athletes’ physical condition and gender will affect the choice of the function of shoes, and the choice of shoes will also affect the athletes when coping with different activities [12]. In Lam’s study, it was found that different heel curve designs affect the performance of good badminton players and their GRF and vertical load rates, with the potential risk of causing related injuries [13]. In recent years, in addition to sport-specific footwear, functional insoles, which provide anti-vibration, anti-slip, and arch support, have led many athletes to wear arch support insoles (ASI) as foot orthoses to improve performance and reduce sports injuries. Huang et al. [14] used ASI to intervene in baseball pitchers’ throwing motion and found that the striding foot and knee stability improved when the subjects wore ASI. ASI is also effective in reducing the impact on the ground, thus avoiding lower limb injuries and increasing lower limb stability [15,16,17]. In other sports, insoles are also effective in reducing lower extremity injuries [18,19].

The objective of this study was to examine the differences in biomechanical parameters and sports-specific performance of lower limbs between ASI and flat insoles (FLI) when performing net strides. It was hypothesized that wearing ASI would be superior to FLI in terms of reducing injury risk and sport-specific performance of lower extremities.

## 2. Materials and Methods

### 2.1. Participants

In the present study, 18 college badminton team members training 36 h a week were recruited as subjects (11 males and 7 females; age: 20.31 ± 1.21; 63.22 ± 7.47 kg; 171.23 ± 13.52 cm). All subjects were right-handed and stride with their right foot. None of the subjects had any injury to the upper or lower limbs that affected their movements within the experimental period or 6 months period before the study.

### 2.2. Procedures

After explaining the purpose and procedure of the experiment and filling out the basic information and consent form, the subjects began to warm up. Subjects wore their familiar badminton shoes and socks. With a wireless real-time motion capture sensor system (MVN Awinda Xsens Technologies BV, Enschede, The Netherlands, 60 Hz), 17 inertial sensors were placed each on the head, sternum, and sacral vertebrae, as well as each on the left and right scapulae, upper arm, forearm, opisthenar, thigh, calf, and instep, to capture kinematics data from the net stride. The force plate (AMTI Advanced Mechanical Technology Inc., Watertown, MA, USA, 1200 Hz) was used to capture kinetics data, and the signal capture box of the wireless motion capture system was applied to capture data simultaneously.

**Figure 1:**
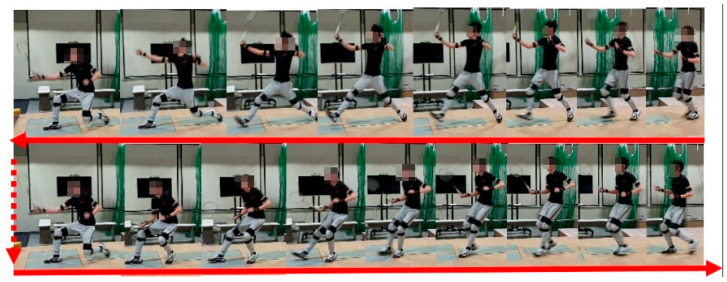
Consecutive net stride tests.

**Figure 2:**
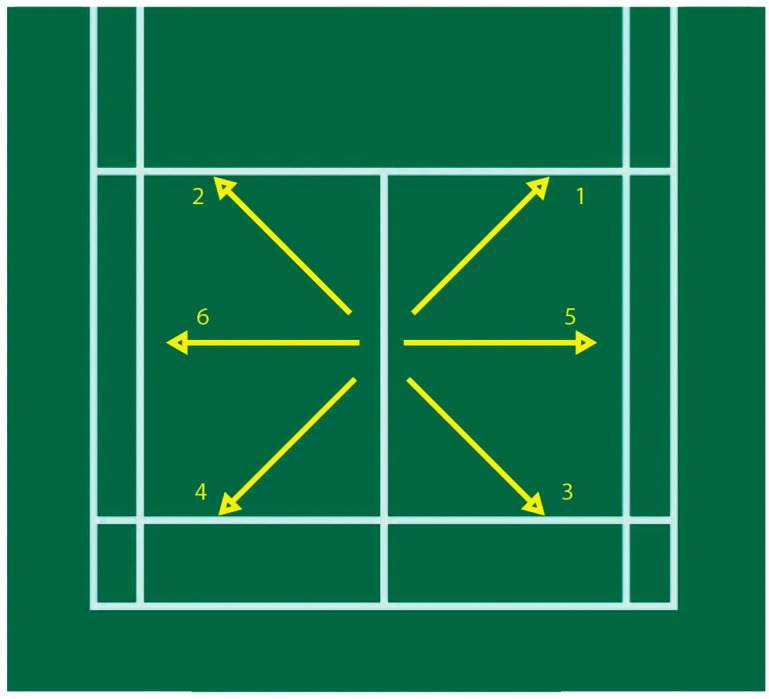
Consecutive six-point footwork tests (1–6 represents the sequence of the six-point).

**Figure 3:**
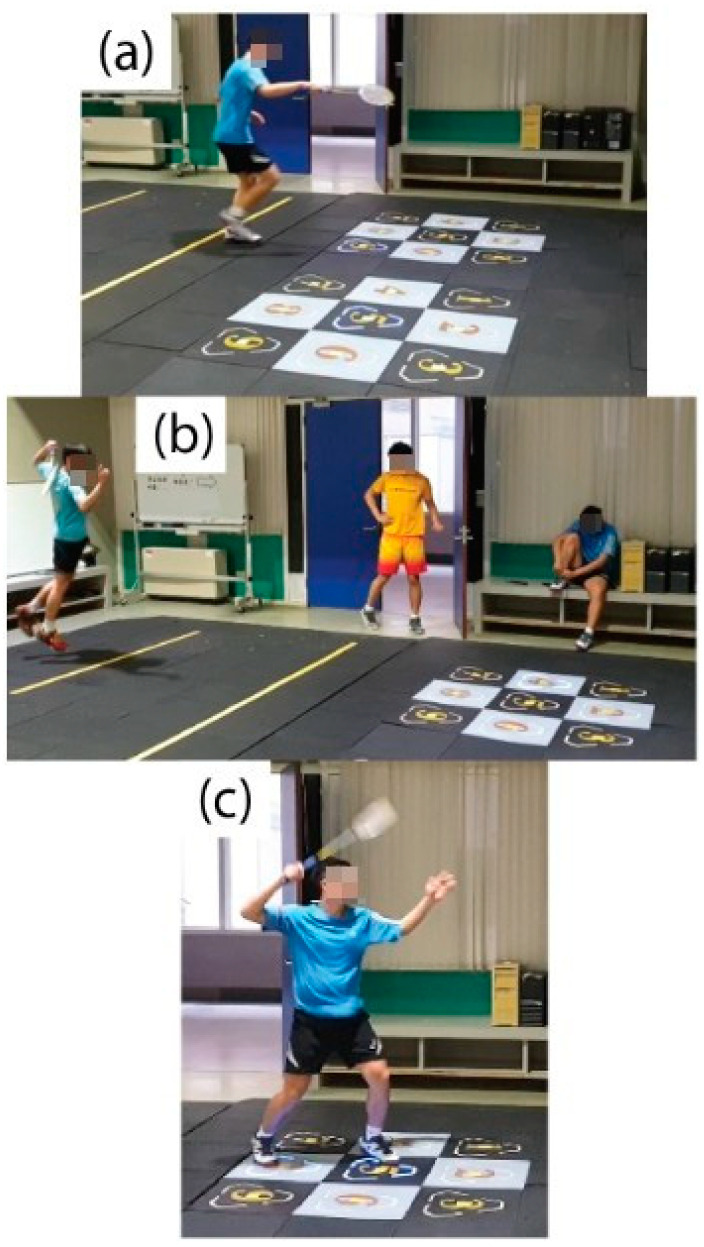
Movement test: ***(*a**) Retrieve/stroke the ball at the left and right net; (**b**) Smash and retrieve/stroke the ball at the net; (**c**) Smash at the front and back crossover step.

The experiment was performed using three tests in three days, each at least 24 h apart. The test was conducted when subjects randomly wore either ASI or FLI with a five-minute break between the two insole tests. The following is the content of the test:

1. Test 1: Choose one day in the sports biomechanics laboratory to perform ten consecutive net stride tests (Figure 1); if there are five accumulated failures (step out of the force plate), cancel the test and re-administer the test. After excluding the first successful net stride, take four consecutive successful movements for analysis.
2. Test 2: Choose one day to perform three consecutive six-point footwork tests on the badminton court (Figure 2) and measure the completion time.
3. Test 3: Choose one day to perform a special movement test on a Sudoku intelligent sports mat. (1) Retrieve/stroke the ball at the left and right net (Figure 3a): the starting mat is 110 cm away from the target mat. Start sprinting to the right target mat and use right foot to do the net retrieve/stroke action at the random lighted place of the mat, run backward to the starting mat, go to the left target mat and step on the lighted place, then return. Repeat to the left and right mats 15 times each, 30 times in total, and measure the completion time. (2) Smash and retrieve/stroke the ball at the net (Figure 3b): the starting mat is 270 cm away from the target mat. Start sprinting after performing the smashing action, use the right foot to perform the net retrieve/stroke action at the random lighted place of the mat, run backward to the starting mat, and smash and retrieve/stroke the ball at the target mat again; repeat 15 times in total, and measure the completion time. (3) Smash at the front and back crossover step (Figure 3c): do this continuously; each time the smash is completed, the right foot steps onto the No. 5 mat, time 30 s, and measure the number of times completed.

### 2.3. Data Analysis

The data were analyzed by MVN (MVN Awinda Xsens Technologies BV, Enschede, The Netherlands) using a low-pass Butterworth filter to process the raw signal data of the sensor and capture the parameters during the net stride support phase [20,21]. The net stride support phase encloses the moment from when the foot touches the force plate to the moment the foot leaves the force plate, using the vertical GRF of the force plate of 20 Newton as the cut-off value. The data collected by MVN includes right hip, knee flexion, foot plantar flexion, foot inversion angle during the landing moment, peak right hip, knee flexion, foot plantar flexion, and foot inversion angles during the support phase.

MotionMonitor uses a low-pass Butterworth filter to process the original force plate signal data and then extracted parameters [22], including the peak GRF and load rates in vertical, horizontal front, and back directions during support and the foot contact time on the force plate as the net stride support time. The data of MVN and GRF uses the moment of the net stride touchdown as the synchronization point.

The performance data of badminton-specific movements included the time of the six-point footwork, the time of retrieving/stroking the ball at the left and right net, the time of smashing and retrieving/stroking the ball at the net, and the number of smashes at the front and back crossover step.

### 2.4. Statistics

The Wilcoxon sign rank test was adopted to compare the differences between ASI and FLI using SPSS statistical software (SPSS 26, Inc., Chicago, IL, USA); the statistical significance was set at α = 0.05. Effect size (ES) was calculated using Cohen’s d.

## 3. Results

In terms of the joint angle during the support phase of test 1, we found that the peak right hip flexion angle was significantly greater with ASI than FLI (63.09 ± 10.70; 60.08 ± 13.82; *p* = 0.028), while the peak right foot inversion angle was significantly smaller with ASI than FLI (20.68 ± 7.87; 23.85 ± 8.11; *p* = 0.013). No significant differences were observed between ASI and FLI for the joint angle at the moment of landing in the GRF data from test 1 and the performance data from tests 2 and 3 (Table 1).

## 4. Discussion

The main finding was that wearing ASI significantly increased the peak hip flexion angle and decreased the peak foot inversion angle of the supporting foot during the landing of the net stride compared to FLI. However, there was no significant difference in the performance of badminton-specific movements.

With the use of ASI, the peak hip flexion angle of lower extremities during stride landing support was significantly increased, i.e., the hip was in a more flexed position for stride support. Previous studies have shown that although a stiffer lower extremity posture at the moment of landing helps to maintain postural stability quickly and urgently [23], there is a risk of injury to the anterior cruciate ligament [24]. The use of hip flexion can mitigate the impact of GRF [25,26,27]. Blackburn and Padua [28] noted that increasing the hip and knee flexion angle during landing reduces the risk of ACL injury. This may indicate a lower overall body center of gravity, and greater use of hip flexion to support the cushioning of the subject in response to his or her net stride, deceleration and abrupt stop, and directional change, thus improving stability.

The lower extremity is exposed to approximately 2.2 BW of vertical GRF and 1.2 BW of horizontal GRF during a net stride. The GRF results in the present study agree with those of Kuntze’s study of badminton striding [29]. The player strides forward rapidly, stops sharply, and then backs up quickly, and this load is primarily transferred to the foot. As the impact usually occurs between the foot and the ground, repeated impacts increase the risk of cumulative lower extremity injury. The findings did not show significant differences between the GRF and its load rate, but both tended to decrease when wearing ASI, which may be more beneficial over time.

This study revealed that the peak foot inversion angle was significantly reduced during the net stride support phase when wearing ASI. The ankle often moves with plantar flexion and internal rotation, if too much foot inversion coincides, the athlete will be at risk for ankle sprain [30]. A greater angle of foot inversion increases the risk of a lateral ankle sprain. Injuries to the lateral ankle ligaments often result from sudden and unexpected excessive foot inversion movement [31]. The badminton-specific striding movement is a step forward and then a quick step backward, so it is important to maintain foot stability during this rapid movement.

ASI can provide athletes with more knee and ankle stability [14]. With this foundation, the athlete can move forward by generating greater hip flexion and having a greater stride while maintaining balance and cushioning. This may increase the efficiency of the athlete’s movement on the court. However, there was no significant difference in time found in the six-point footwork test of this study. The athletes might be fully prepared psychologically because the content of the test has been informed to the athletes in advance. As a result, the athletes might pursue efficiency and experimental completion instead of completely pursuing speed when testing. In future research, a stride test with sudden responses can be designed to study the athlete’s response and movement speed.

There was a tendency to improve the performance of badminton-specific movements when wearing ASI in the current study. Players were able to reduce the seconds to perform the six-point footwork, retrieve/stroke the ball at the left and right net, smash and retrieve/stroke the ball at the net, or increase the number of smashes at the front and back crossover step in 30 s, but neither reached statistical significance. It was assumed that a high level of technical maturity or adaptation to the insoles in the subjects of this study may have prevented ASI from showing its benefits in this one-time, short-term performance test for badminton-specific movements. It is worthwhile to further investigate whether the physiological benefits of ASI will be reflected in the physiological savings if the athletes wear ASI for a long time for sport-specific training. 

This study has the following limitations: The experimental procedure was designed through a program, and the subjects were in anticipation when they performed it and did not randomly react to the scene during the competition, possibly failing to present the real athletic situation of the athletes. Subjects wore ASI only during the experiment, and the design of the experiment only considered immediate effects. The arch type of the subjects was not grouped; perhaps differences in arches may have affected the results. Only the six-point footwork step was conducted on the badminton court in the experiment. When the experiment was conducted on the force plate and the smart sports mat in the laboratory, the softness of the ground material and the friction force differed from those on the real badminton court, thereby affecting the athletes’ performance.

## 5. Conclusions

The important effects of ASI on the net stride in badminton are reducing the varus angle of the supporting ankle and increasing the hip flexion angle. The actual ascertainment of injury prevention and performance enhancement would be required to enable the practical use of ASI over a long period.

## Figures and Tables

**Table 1:** Biomechanical parameters of consecutive net stride tests and performance.

Parameters	FLI	ASI	%	*p*	ES
**Kinematics (Test 1)**					
Support time (s)	0.60 ± 0.11	0.59 ± 0.15	−1.7	0.506	0.16
*The moment of landing*					
RhipF (deg)	42.58 ± 10.09	44.75 ± 14.64	5.1	0.17	0.32
RkneeF (deg)	31.37 ± 17.22	35.85 ± 20.19	14.3	0.523	0.15
RfootI (deg)	13.97 ± 7.13	12.46 ± 4.8	−10.8	0.326	0.25
RfootPF (deg)	12.67 ± 9.56	5.37 ± 15.38	−57.6	0.272	0.32
*Peak value during support phase*					
RhipF (deg)	60.08 ± 13.82	63.09 ± 10.70	5.0	0.028 *	0.52
RkneeF (deg)	58.79 ± 10.86	60.41 ± 13.87	2.8	0.372	0.21
RfootI (deg)	23.85 ± 8.11	20.68 ± 7.87	−13.3	0.013 *	0.59
RfootPF (deg)	36.07 ± 15.94	31.1 ± 12.38	−13.8	0.332	0.24
**Kinetics (Test 1)**					
PvGRF (BW)	2.16 ± 0.30	2.16 ± 0.32	0	0.948	0.02
PhGRF (BW)	1.23 ± 0.24	1.22 ± 0.20	−0.8	0.845	0.05
VLR (BW/s)	26.12 ± 12.69	26 ± 12.26	−0.5	0.828	0.05
HLR (BW/s)	16.28 ± 8.28	15.82 ± 6.96	−2.8	0.711	0.09
**Performance**					
Test 2 (s)	36.97 ± 3.00	36.34 ± 3.12	−1.7	0.210	0.29
Test 3 (1) (s)	63.85 ± 13.4	62.18 ± 11.47	−2.7	0.372	0.21
Test 3 (2) (s)	53.31 ± 8.53	52.06 ± 10.51	−2.3	0.777	0.06
Test 3 (3) (times)	30.6 ± 2.96	31.5 ± 3.96	2.9	0.184	0.31

* significant difference found between FLI and ASI. *p* < 0.05. RhipF: Right hip flexion; RkneeF: Right knee flexion;RfootI: Right foot inversion; RfootPF: Right foot plantar flexion; PvGRF: Peak vertical GRF; PhGRF: Peak horizontal GRF; VLR: Vertical loading rate; HLR: Horizontal loading rate. Positive percentage values indicate an increase relative to FLI, and negative values indicate a decrease relative to FLI.

## Data Availability

The data that support the findings of this study are available from the corresponding author upon reasonable request.
